# Preferences and perceived barriers for internet-based treatment among adolescents with anxiety or depressive disorders: A qualitative study

**DOI:** 10.1016/j.invent.2024.100770

**Published:** 2024-09-05

**Authors:** J. Emmelkamp, M.A. Wisman, M.H. Nauta, N.I.E. Van Rijn, J.J.M. Dekker, C. Christ

**Affiliations:** a Arkin Mental Health Care, Department of Research, Amsterdam, the Netherlands; b Vrije Universiteit Amsterdam, Department of Clinical, Neuro and Developmental Psychology, Amsterdam Public Health research institute, Amsterdam, the Netherlands; c Department of Youth and Family, Arkin Mental Health Care, Amsterdam, the Netherlands; d University of Groningen, Department of Clinical Psychology and Experimental Psychopathology, Groningen, the Netherlands; e Accare Child Study Center, University Center for Child and Adolescent Psychiatry, Groningen, the Netherlands; f GGZ inGeest Mental Health Care, Amsterdam, the Netherlands; g Vrije Universiteit Amsterdam, Department of Clinical Psychology, Faculty of Behavioral and Movement Sciences, Amsterdam Public Health research institute, Amsterdam, Netherlands; h Amsterdam Public Health Research Institute, Amsterdam UMC, VU University Medical Center, Department of Psychiatry, Amsterdam, the Netherlands

**Keywords:** Adolescents, Anxiety, Depression, Focus group, Qualitative study, Ehealth

## Abstract

**Background:**

Over the past two decades, the development of internet-based treatments for adolescents with anxiety and depressive disorders has advanced rapidly. To date, adolescents' preferences and perceived barriers for internet-based treatment remain largely unknown, especially in clinical samples. Therefore, this study explored the preferences and perceived barriers of adolescents with anxiety or depression regarding internet-based treatment.

**Methods:**

This qualitative study included 21 adolescent patients with anxiety or depressive disorder, and varied levels of experience with internet-based treatment. Two focus groups (N_1_ = 5, N_2_ = 6) and semi-structured interviews (*N* = 10) were conducted, recorded, transcribed, and analyzed using a reflexive thematic analysis approach.

**Results:**

The thematic analysis yielded five main themes, and 12 subthemes. The main themes were: independence, accessibility, content, therapist contact, and appearance. Adolescents highlighted self-direction as a benefit of internet-based treatment, and motivational challenges as a drawback. They found internet-based interventions convenient and particularly fitting for implementation during waiting periods before formal treatment. Guided interventions were preferred over mere self-help. Furthermore, adolescents stressed the importance of a clear, organized design, and recommended accessibility on both mobile phones and computers.

**Conclusion:**

Findings provide a clear overview of the needs and preferences of adolescents with anxiety or depressive disorder regarding internet-based treatment. To address their diverse needs, internet-based interventions should be tailorable, should incorporate therapist guidance, and should already be available during the treatment waiting period. Results of this study can guide the development and implementation of new internet-based interventions, and may thereby help to further optimize their uptake among adolescent patients.

## Highlights

• Treatment-seeking adolescents' preferences for internet-based treatment were explored.
• Self-direction was considered a benefit, and motivational challenges were a drawback.
• Adolescents preferred guided, accessible interventions with a clear design.
• Personalizing interventions is important to align with different needs and desires.
• Study results can be used for the development of new internet-based interventions.

## 1 Introduction

Anxiety and depressive disorders are common in adolescence (Polanczyk et al., 2015). Both disorders cause severe distress and have a negative impact on adolescents' social functioning, educational achievements, physical health, and quality of life (de Lijster et al., 2018; Jaycox et al., 2009). Moreover, anxiety and depressive disorders can persist into adulthood and predict a variety of other adult mental health disorders (Essau et al., 2014). Even though the disease burden of both disorders is high, many youths do not receive treatment (Mojtabai et al., 2016). Adolescents often face barriers in seeking help for mental health problems, such as stigma and embarrassment, inaccessibility, and long waitlists (Gulliver et al., 2010; MacDonald et al., 2021).

Internet-based treatment (i.e., treatment delivered via the internet on smartphone apps or websites) has emerged as an alternative to face-to-face therapy that may help overcome these barriers (e.g., Andrews et al., 2018; Batterham et al., 2015). Several meta-analyses have shown that internet-based treatments are effective for adolescents with anxiety and depressive disorders (e.g., Christ et al., 2020; Grist et al., 2018). Further, internet-based treatment appears to be cost-effective for adults with anxiety or depressive disorder (Mitchell et al., 2021). Moreover, these treatments are found to be a feasible substitute for traditional face-to-face therapies for adolescents due to their compatibility with their digital lifestyle, time efficiency, and convenience (Andersson and Titov, 2014; Domhardt et al., 2018).

Despite increased interest in internet-based treatment for adolescent mental health issues, several challenges persist. Internet-based treatments generally have lower uptake and higher dropout rates than face-to-face treatments, particularly for self-help interventions without therapist support (Fleming et al., 2018; Richardson et al., 2010). These factors form important barriers for the broad implementation of internet-based treatment into clinical practice, which has proven difficult thus far (Batterham et al., 2015). Understanding adolescents' preferences and perceived barriers regarding internet-based treatment is needed to improve its uptake. A qualitative study conducted by Mar et al. (2014) on a non-clinical sample of adolescents experiencing suicidal ideation found that internet-based interventions should be user-friendly, interactive, and include guidance. Further, some studies have investigated youths' perspectives on specific interventions through co-design to increase engagement (e.g., Ludlow et al., 2023). However, adolescents' perspectives on internet-based treatment in general remain largely unknown, particularly in clinical samples with anxiety or depressive disorder.

### 1.1 Current study

The objective of this qualitative study is to increase insight into preferences and perceived barriers for internet-based treatment among clinically referred adolescents with anxiety or depressive disorder. More specifically, we will examine their views and experiences regarding advantages and disadvantages, important functions, guidance, and appearance of internet-based treatment.

## 2 Methods

### 2.1 Participants

All 39 participants from a larger pilot study (Wisman et al., 2023) were invited to this qualitative study. 21 (53.8 %) adolescents with anxiety or depressive disorders, referred to mental health care, participated in focus groups and semi-structured interviews. Participants did not differ on any demographic variable at baseline from other participants in our pilot study, which aimed to examine the acceptability and feasibility of an add-on internet-based emotion regulation training (ERT) for adolescents with depressive and/or anxiety disorder. Inclusion criteria were: aged 13–18 years at baseline; diagnosed with depressive or anxiety disorder; enrolled for treatment at Arkin in Amsterdam. A more detailed description is given elsewhere (Wisman et al., 2023).

Table 1 shows the characteristics of the participants of the current qualitative study. In total 21 participants, aged 15–20 (M = 17.1, SD = 1.4), were included. Around half of the adolescents had experience with any form of internet-based treatment for psychological problems.

**Table 1:** Demographic variables.

	Total sample (*N* = 21)
Age in years, mean (*SD*)	17.1 (1.4)
Sex, *n* (%)	
Female	17 (81.0)
Male	4 (19.0)
Condition, *n* (%)	
ERT	9 (42.9)
Control	12 (57.1)
Experience with internet-based treatment, *n* (%)	
No experience	7 (33.3)
Little experience (1–3 times logged in)	4 (19.0)
Experience (>3 times logged in)	10 (47.6)
Primary diagnosis, *n* (%)	
Depressive disorder	13 (61.9)
Anxiety disorder	8 (38.1)
Comorbid depression/anxiety, *n* (%)	7 (33.3)
Ethnicity, *n* (%)	
Dutch	14 (66.7)
2nd generation Western	3 (14.3)
2nd generation Non-Western	4 (19.0)

### 2.2 Ethics

All participants provided written informed consent as well as the parents of the participants under 16. The Medical Research Ethics Committees United (MEC-U) has approved the study.

### 2.3 Data collection

Qualitative data were collected between December 2020 and November 2021 through focus groups and semi-structured interviews, which took place through videoconference due to COVID-19 restrictions. When contacted, the topics and approximate duration of the focus groups and the interviews were explained. Participants received a gift card of 25 euros for their time and effort.

**Fig. 1:**
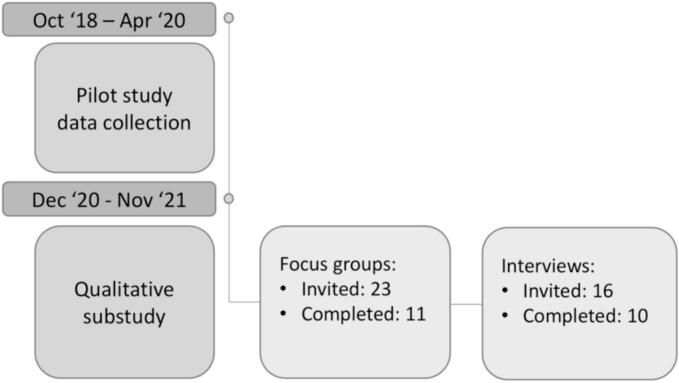
Flow chart of the study.

Two focus groups were conducted with *N* = 5 and *N* = 6 participants, followed by semi-structured interviews with N = 10 others (see Fig. 1). Initially, we aimed to form two focus groups of six participants each by inviting 23 randomly selected individuals, resulting in 11 attending and one no-show. As data saturation was not reached, we sought deeper insights into the themes by contacting the remaining 16 participants from the pilot study. Ten agreed to participate in additional semi-structured interviews. Reasons for declining included being too busy (*N* = 4) or not interested (N = 4). Others provided no reason or were unreachable (*N* = 9). One participant agreed at first, but was unreachable afterward.

#### 2.3.1 Focus groups

The duration of both focus groups was 90 min. The topic list of the focus groups was based on previous research (e.g., Liverpool et al., 2020; Mar et al., 2014) and the current research question, and was used to roughly structure the focus groups (See Appendix A). Open-ended questions were used to initiate discussions and offer room for deviation into other topics, and follow-up questions were used to gain a more profound comprehension of the participants' perspectives (Rubin and Rubin, 2005). The discussions were moderated by a clinical psychologist/researcher (MW) and a child psychologist/researcher (JE).

#### 2.3.2 Semi-structured interviews

To add more depth to the topics discussed in the focus groups, complementary individual semi-structured interviews were held, which lasted 24 to 57 min. The topic list was similar to the one used in the focus groups, but some additional questions were added based on outcomes of the focus groups. Each interview was conducted by a child psychologist/researcher (JE), and a research assistant was present during most interviews.

### 2.4 Data analyses

All audio-files were transcribed anonymously and texts were analyzed using reflexive thematic analysis (TA; Braun and Clarke, 2006). TA is described as “a method for identifying, analyzing and reporting patterns (themes) within data” (Braun and Clarke, 2006; page 79). In reflexive TA, meaningful themes are identified and analyzed by the use of structured steps (Braun and Clarke, 2006). It was chosen for its flexibility, systematic approach, and ability to identify and analyze patterns within qualitative data, making it well-suited to explore the perspectives and experiences shared by the adolescents. First, one researcher (JE) became familiar with the data and generated initial codes using MAXQDA 2022 (VERBI software, 2021). Subsequently, two researchers (JE, MW) developed the coding framework and coded the data. Third, two researchers (JE, MW) identified themes, which they reviewed in relation to the codes and the data set. Finally, they differentiated the themes into subthemes. The analyses were reviewed, revised, and discussed with CC and MN. Disagreement between authors was resolved through comments, feedback, and in-depth discussions of the themes until consensus was reached. Descriptive analyses of quantitative data were performed with IBM SPSS Statistis, version 28.0.

## 3 Results

The reflexive TA resulted in five main themes, with a total of 12 subthemes (see Table 2), which are discussed below.

**Table 2:** Themes and subthemes.

Themes	Subthemes
Independence	Advantages of independence
	Disadvantages of independence
Accessibility	Convenience
	Availability
Content	Psychoeducation
	Acquiring new skills
	Exercises
	Personalization
Therapist contact	Therapeutic relationship
	Guidance
Appearance	Design
	Device

### 3.1 Theme (1) Independence

The first theme ‘independence’ included the subthemes: ‘advantages of independence’ and ‘disadvantages of independence’. See Table 3 for illustrative quotes per subtheme.

**Table 3:** Theme (1) Independence, illustrative quotes.

Subtheme		Illustrative quotes
Advantages of independence	1	Participant 17 SI, E: *“I think it works better to figure out what works best for you. You can figure it out for yourself, without someone telling you what's better for you.”*
	2	Participant 14 SI, E: *“I think the most important function is that you can decide for yourself which parts you want to follow or not, that it is quite self-directed.”*
Disadvantages of independence	3	Participant 7 FG, E: *“The fact that you can just do it at home means that you really have to motivate yourself to do it and that it might feel a bit like homework.”*
	4	Participant 1 FG, E: *“I'm probably just not thinking about it very much. And then it's probably just not on my mind and that's why I just forget it.”*
	5	Participant 13 SI, E: *“If you have depression, it's just really hard to take a step to do something. Because everything just feels like a lot of stress and a lot of time pressure.”*

Note: E: Previous experience with internet-based treatment; NE: No experience with internet-based treatment; SI: Semi-structured interview participant; FG: Focus group participant.

#### 3.1.1 Advantages of independence

Several advantages of the independent nature of internet-based treatment were brought up: adolescents liked being able to work on their mental health issues on their own^1^*.* The independent nature of internet-based treatment would provide them more self-direction over their therapy compared to regular face-to-face treatment. Moreover, having the option to choose specific aspects of an intervention was mentioned as a benefit^2^.

#### 3.1.2 Disadvantages of independence

On the other hand, adolescents also expressed challenges related to independence in internet-based treatment. The experience of completing treatment independently was linked to the feeling of having to complete homework^3^. Adolescents noted they may struggle to stay engaged and motivated when completing internet-based treatment, without the same level of encouragement often provided in face-to-face settings^4,5^. Another concern raised was the risk of forgetting or postponing to log in. Some preferred the structure of face-to-face therapy, with set appointments and follow-up from a therapist.

### 3.2 Theme (2) Accessibility

The second theme was ‘accessibility’, with two subthemes: ‘convenience’ and ‘availability’ (see Table 4).

**Table 4:** Theme (2) Accessibility, illustrative quotes.

Subtheme		Illustrative quotes
Convenience	6	Participant 7 FG, E: *“You don't have to go all the way there, and that it's much more practical. You can do it on your computer at home, which takes less time.”*
	7	Participant 21 SI, NE: *“I personally prefer to have therapy from home because I feel safe there and I feel comfortable there. For me, it's sometimes very difficult to make a lot of effort to get out of bed or leave the house.”*
Availability	8	Participant 13 SI, E: *“The accessible offering of last-minute help. If you think, for example, ‘Oh, I have a test this afternoon and I always get really stressed before tests,’ then you can go to that app and you can still do a zen exercise or watch that video that talked about it. It's a kind of last-minute help that a therapist cannot offer.”*
	9	Participant 12 SI, E: *“Before I could talk to someone, I was on a kind of waiting list, so then you could do it, for example [internet-based treatment]. At that time, I didn't know what was wrong with me, so I was already stressing out about what was happening to me. (…) I think that would be very good.”*

Note: E: Previous experience with internet-based treatment; NE: No experience with internet-based treatment; SI: Semi-structured interview participant; FG: Focus group participant.

#### 3.2.1 Convenience

Adolescents emphasized the convenience and practicality of internet-based treatment. This form of treatment would be less time consuming and suits their busy schedules, due to the absence of physical appointments and travel time^6^. Additionally, some adolescents stressed the convenience of staying at home, where they feel secure^7^. Others mentioned that avoiding the additional effort of leaving the house enables more focus on the therapy itself.

#### 3.2.2 Availability

Adolescents mentioned the benefits of availability of treatment: receiving help whenever needed. Internet-based treatments can provide immediate support, which traditional face-to-face therapy cannot always provide^8^. Some adolescents experience long waiting lists before receiving face-to-face treatments, causing additional stress and anxiety. Internet-based treatment may be beneficial during this waiting time^9^.

### 3.3 Theme (3) Content

The third theme ‘content’ included the following subthemes: ‘psychoeducation’, ‘acquiring new skills’, ‘exercises’, and ‘personalization’ (see Table 5).

**Table 5:** Theme (3) Content, illustrative quotes.

Subtheme		Illustrative quotes
Psychoeducation	10	Participant 18 SI, NE: *“If you're struggling with something or something is going on, you can open the module and look it up.”*
	11	Participant 12 SI, E: *“Well, I have seen videos of people who have a little bit the same thing as I have, well that helps a bit because then you know you're not alone.”*
	12	Participant 12 SI, E: *“And, for example, explaining to my parents what's going on. They don't need to know exactly how I feel or whether I'm super depressed this week. I don't want to discuss that with them. But they do need to know what's going on, you know.”*
Acquiring new skills	13	Participant 15 SI, E: *“That you learn something from it and learn some ways to deal with it. That's the most important thing for me.”*
	14	Participant 17 SI, E *“Just giving you extra skills to deal with depression or anxious symptoms and stuff. That you know a bit more about what to do.”*
Exercises	15	Participant 16 SI, E: *“The more exercises, the better, but not that you have to do all of them anyway.”*
	16	Participant 14 SI, E: *“I think practicing in real life is better because then you can learn more about your situation and how to deal with it.”*
	17	Participant 18 SI, NE: *“Homework is good because you are still working on it afterward.”*
	18	Participant 10 FG, NE: *“I think that homework will work counterproductively. Most people who have problems and need therapy, at least a large part of them, I think they have a lot of trouble with school and stuff. And I think that homework is very much associated with that and has a very negative impact.”*
Personalization	19	Participant 14 SI, E: *“I actually got quite rebellious about it myself because it was all: ‘you have to do this’ and ‘you have to do that’. And then I thought: ‘yes, but maybe I don't want to do that at all? Maybe it doesn't work for me?’. It is quite a standard module that you follow, and it's not really tailored to your specific needs.”*
	20	Participant 8 FG, E: *“It's much less helpful for me to have a platform that is very specific. I would rather have a very large platform. So people don't feel like they're being put into boxes or that it's forced upon them, so that you have all the freedom to choose.”*

Note: E: Previous experience with internet-based treatment; NE: No experience with internet-based treatment; SI: Semi-structured interview participant; FG: Focus group participant.

#### 3.3.1 Psychoeducation

Having easily accessible information was stressed as one of the benefits of internet-based treatment^10^. The online psychoeducation could have the added advantage of reducing feelings of isolation for adolescents. Recognition in the description of mental health problems, experience stories, and videos could provide a sense of community for them^11^. Furthermore, one adolescent noted that it would be beneficial to provide parents online psychoeducational resources regarding their child's mental disorder^12^.

#### 3.3.2 Acquiring new skills

Adolescents expressed that one of the main functions of this type of treatment was to acquire new skills: provide practical tools and strategies to help them cope with their mental health problems^13,14^. This could contribute to an overall sense of well-being.

#### 3.3.3 Exercises

Adolescents frequently acknowledged exercises as an important aspect of internet-based treatment, but preferred them to be optional. They valued flexibility and indicated that they would feel constrained if completion of all exercises was required for program advancement. While some adolescents preferred exercises within the platform, others suggested practicing in real-life could be more effective^15,16^. Regarding homework exercises, some found these beneficial for addressing issues outside sessions, while others disliked them due to associations with school-related stress^17,18^.

#### 3.3.4 Personalization

Some of the adolescents with prior experience with internet-based treatment mentioned that standard modules were not tailored to their needs^19^. Rather than offering restrictive, obligatory exercises, they suggested that a broad range of exercises should be available, leaving room for tailoring to the individual's needs and pace^20^.

### 3.4 Theme (4) Therapist contact

For the fourth theme ‘therapist contact’, two subthemes emerged: ‘therapeutic relationship’ and ‘guidance’ (see Table 6).

**Table 6:** Theme (4) Therapist contact, illustrative quotes.

Subtheme		Illustrative quotes
Therapeutic relationship	21	Participant 9 FG, NE: “*I am definitely against it [internet-based treatment]. It seems very inhumane to me.”*
	22	Participant 14 SI, E: *“It feels safer and you don't have to share all your feelings with a person, so you are less likely to experience a form of rejection or something like that.”*
	23	Participant 18 SI, NE: *“I only have trust once I've seen someone, once I have an image of someone.”*
	24	Participant 10 FG, NE: *It's just a different kind of relationship. I've had quite a few online friends who were really good friends of mine.”*
Guidance	25	Participant 19 SI, NE: *“I think you'd still want the opportunity for feedback. […] If you don't know you're doing it wrong, you'll keep doing it the same way.”*
	26	Participant 6 FG, E: *“If you fill something out online, you should also receive online feedback.”*
	27	Participant 9 FG, NE: *“I think if you get feedback in person, it's more valuable and sticks better with people, and it doesn't feel very formal, which has less of an effect.”*

Note: E: Previous experience with internet-based treatment; NE: No experience with internet-based treatment; SI: Semi-structured interview participant; FG: Focus group participant.

#### 3.4.1 Therapeutic relationship

Most adolescents felt that internet-based treatment lacks the personal connection that comes with face-to-face therapy. By some, internet-based therapy was perceived as being too mechanical and unnatural to replace face-to-face therapy^21^. Interpreting tone and emotion was perceived as more challenging in an online environment.

Most adolescents mentioned that internet-based therapy could provide a sense of safety and security that face-to-face therapy may lack. Anonymity and physical distance provided by the screen may make it easier for people to express themselves^22^. The online nature would allow for a more thoughtful and edited expression of thoughts and emotions. This was perceived as particularly beneficial for those with difficulties expressing themselves verbally, such as adolescents with anxiety.

Some stated that building a therapeutic relationship in an internet-based treatment was not possible for them, or that the bond would not be as strong as in face-to-face therapy^23^. Others mentioned that it would be possible, similar to forming close relationships with online friends^24^.

#### 3.4.2 Guidance

Almost all adolescents preferred guided internet-based treatment over self-help^25^. Feedback provides a sense of human contact, can provide a framework, helps them track their progress in the treatment, and informs them whether they are on the right track or not. Some preferred to receive online feedback through a chat function within the online platform or email, while others preferred face-to-face feedback^26,27^.

### 3.5 Theme (5) Appearance

The fifth theme ‘appearance’ included the following subthemes: ‘design’ and ‘device’ (see Table 7).

**Table 7:** Theme (5) Appearance, illustrative quotes.

Subtheme		Illustrative quotes
Design	28	Participant 11 FG, NE: *“I would probably want it to be very organized. That it's very easy to find where everything is, so it's very clear which sessions are where and how to get there and what the session is about”*
	29	Participant 19 SI, NE: *“If it's all dark colors, it can be depressing. If you're already feeling depressed, that doesn't seem very helpful.”*
	30	Participant 21 SI, NE: *“I would make it customizable. So if a person has a certain page on an account, they can customize it with their own colors, fonts, backgrounds, profile photo.”*
Device	31	Participant 13 SI, E: *“If I were in public transportation or I had a moment, then I would open the app more quickly. (…) your phone is just very accessible for that.”*
	32	Participant 13 SI, E: *“It's nice that it's an app. Where if you're just logged in, you stay logged in, for example, and you can also do it on your phone.”*
	33	Participant 14 SI, E: *“A computer or laptop is just more convenient. It's more organized and it's bigger, so you can read and hear things better.”*

Note: E: Previous experience with internet-based treatment; NE: No experience with internet-based treatment; SI: Semi-structured interview participant; FG: Focus group participant.

#### 3.5.1 Design

Adolescents brought up the importance of having a clear, organized design for the internet-based platform, which should be easy to follow and navigate through^28^. The majority of adolescents preferred softer, more calming colors, such as pastels or muted natural colors. Specifically red and black were mentioned to be avoided, being associated with failure or negativism, and a depressed mood, respectively^29^. Some adolescents desired the ability to personalize and customize the look of the internet-based platform^30^.

#### 3.5.2 Device

Some adolescents found it more convenient to use an app on their mobile phone, which allows for setting reminders and engaging wherever and whenever they have a spare moment^31,32^. Others preferred following internet-based treatment from a laptop or computer, which would be more organized, easier to read and write on, and may be less distracting than a mobile phone^33^.

## 4 Discussion

### 4.1 General findings

This study aimed to explore preferences and perceived barriers for internet-based treatment among adolescents with anxiety or depressive disorder. Focus groups and semi-structured interviews revealed five main themes, which are discussed below.

The first theme that came forward was ‘independence’, with the subthemes ‘advantages of independence’ and ‘disadvantages of independence’. One of the perceived advantages of independence was self-direction over one's therapy, in line with earlier research among youths (Hollis et al., 2017). Perceived disadvantages included the lack of structured appointments, and motivational challenges, especially for depressed adolescents. A review concluded that many children and adolescents with mental health problems do not participate in internet-based treatment due to time constraints (Liverpool et al., 2020). Our study indicates that adolescents with anxiety or depressive disorder may not experience difficulties in “having time”, but rather in “making time” (e.g., self-motivation).

The second theme was ‘accessibility’. The first subtheme ‘convenience’ highlights the time-saving nature and compatibility of internet-based treatment with busy schedules, which is also a commonly cited advantage among adults (e.g., Andersson and Titov, 2014). The second subtheme that came forward was ‘availability’ of internet-based treatment. Adolescents greatly appreciated the idea of starting with internet-based treatment while waiting for face-to-face treatment, which corresponds with professional Dutch guidelines that consider e-health highly suitable for waitlist support among youths (Akwa, 2022). However, the current provision of internet-based interventions for adolescents during the waiting period remains limited. A survey among U.S. therapists indicated that only a minority currently uses internet-based treatment as a waiting list intervention, despite a widespread willingness to do so (Peipert et al., 2022).

The third theme that arose was ‘content’, with subthemes ‘psychoeducation’, ‘acquiring new skills’, ‘exercises’, and ‘personalization’. In line with a recent study (Leigh et al., 2023), adolescents mentioned that psychoeducation could potentially reduce feelings of isolation, for example by experience stories. Additionally, offering practical tools to acquire new skills and thereby increase coping and well-being was considered important. Views on exercises and homework in internet-based treatment varied. Homework engagement is associated with positive clinical outcomes (e.g., Mausbach et al., 2010), and online homework has been suggested as a possible solution to improve engagement (e.g., Bunnell et al., 2021). However, although some of our participants emphasized the importance of exercises or homework, others found them restricting and stressful. In the final subtheme, ‘personalization’, adolescents emphasized the need for tailored modules, aligning with previous research (Garrido et al., 2019; Liverpool et al., 2020).

The fourth theme was ‘therapist contact’, with the subthemes ‘therapeutic relationship’ and ‘guidance’. Adolescents discussed the potential benefits of the online environment in terms of safety and anonymity. This aligns with a meta-analysis into digital health interventions among children and adolescents, in which reduced stigma arose as a benefit (Hollis et al., 2017). However, most adolescents found it difficult to build a therapeutic relationship online, which corresponds to studies showing that adolescents have a strong desire to connect with ‘real people’ and can feel alone in internet-based treatment (Mar et al., 2014; Liverpool et al., 2020). In the present study, a majority preferred guided internet-based treatment over self-help, with some favoring online guidance and a majority leaning toward face-to-face guidance (i.e., blended care). These preferences align with better outcomes of guided internet-based treatment compared to self-help internet-based treatment among adolescents (Grist et al., 2018) and research demonstrating clinician support to enhance adherence to internet-based treatment among adults with anxiety and depression (Richards and Richardson, 2012).

The final theme that arose was ‘appearance’, with the subthemes ‘design’ and ‘device’. Adolescents emphasized the importance of a clear, organized design, in line with a study among a community sample of adolescents and young adults (Garrido et al., 2019). Some mentioned they would prefer to personalize their own internet-based environment, which was also highlighted during the co-design process of a new digital mental health platform for youth (Ludlow et al., 2023). Preferences for devices were diverse, with some favoring mobile phones for convenience, while others preferred laptops or computers for a more formal, organized, and less distracting experience.

### 4.2 Strengths and limitations

Strengths of this study include the combination of focus groups and semi-structured interviews, offering insights into both group dynamics and in-depth individual perspectives. Second, unlike many qualitative studies that focus on the feasibility of specific internet-based interventions, this study takes a broader approach by exploring general views, preferences and barriers to internet-based treatment. Further, the inclusion of participants of a clinical population with common mental disorders and with varied levels of experience with internet-based treatment provides a more comprehensive understanding of the target population. This study also has a number of limitations. The findings may have limited transferability due to the specific context and sample characteristics, which should be considered when applying the results to other settings. Furthermore, a potential selection bias may exist as only adolescents willing to participate were included. Additionally, preconceptions may have influenced the coding process, potentially impacting the confirmability of the results. Further, focus groups and interviews were conducted via videoconference, and while videoconferencing can yield data quality comparable to face-to-face methods, it presents challenges such as technical issues and difficulties in observing non-verbal cues (Almujlli et al., 2022). Finally, while the inclusion of adolescents with diverse levels of experience in internet-based treatment is considered a strength, it may also have introduced unexamined differences in perspectives based on group characteristics.

### 4.3 Recommendations

First of all, preferences differ substantially between adolescents and further research is needed to explore preferences among different subgroups of adolescents, taking into account various patient characteristics. Results of our study show that personalization of interventions is important to align with their different needs and desires. Self-motivation came forward as one of the most important barriers for internet-based treatment. Sustaining participant motivation through strategies, such as additional reminders (e.g., Achilles et al., 2020), is thus of great importance. Further, to alleviate stress, keeping homework exercises optional and encouraging in-session practice is recommended. Another important recommendation is to develop guided forms of internet-based treatment, preferably with at least one face-to-face or videoconference session with a therapist next to online feedback. Considering the divided preferences regarding type of device, internet-based interventions should be compatible on both mobile phones and computers. Furthermore, adolescents suggested already starting with internet-based treatment during the often alarmingly long waiting lists. Currently, internet-based interventions as waiting list care for adolescents is not yet widely disseminated, while these interventions are well-suited for utilization during the waiting period.

### 4.4 Conclusions

In conclusion, developing internet-based interventions for adolescents with anxiety and depressive disorders is a complex task. The findings from this study highlight the need for thoughtful consideration of the unique needs and preferences of this specific target population. The study highlights the importance of personalized, guided internet-based interventions to address adolescents' diverse needs and motivational barriers. Furthermore, it is advisable to create interventions compatible on both mobile phones and computers, and implement them during the waiting period for treatment. The preferences indicated in the current study may enhance the development and implementation of novel internet-based interventions for the treatment of anxiety and/or depressive disorders.

## Funding

The study is funded by 10.13039/501100007502Stichting tot Steun VCVGZ Research (project nr. 270).

## Compliance with ethical standards

The study was approved by the Medical Research Ethics Committees United (MEC-U).

## CRediT authorship contribution statement

JE and MW conducted the data analyses, which were reviewed, revised, and discussed with CC and MN. JE wrote the first draft of the manuscript. CC, MW, MN, NvR and JD provided feedback. JE finalized the manuscript.

## Declaration of competing interest

The authors declare that they have no known competing financial interests or personal relationships that could have appeared to influence the work reported in this paper.

## Appendix A Topic list

Start:

• Introduction of Moderators
• Participant Introductions: Conduct a quick round of introductions among participants.
• Overview of Internet-Based Treatment: Provide a concise explanation of internet-based treatment.
• Demonstration: Present an example of internet-based treatment using the module from the pilot study by Wisman et al. (2023).

a. Advantages and disadvantages

• What advantages does internet-based treatment (for mental health issues) have for you?
  ➔ Why is … an advantage for you?
• What disadvantages does internet-based treatment (for mental health issues) have for you?
  ➔ Why is … a disadvantage for you?

b. Functions

• What is the most important function of an internet-based treatment module for you?
  ➔ Why do you think this is an important function?
• If you were to follow an internet-based treatment for depressive or anxiety complaints, what components would you like to see in it?
• What kind of content or information should be included?
  ➔ What do you consider most important?
  ➔ What would you rather NOT see in an internet-based treatment?
• What are your thoughts on exercises in an internet-based treatment?
  ➔ Can you further explain your view?
• What are your thoughts on homework in an internet-based treatment?
  ➔ Can you further explain your view?

c. Guidance

• What is your view on guidance in internet-based treatment?
• Would you like to receive feedback from a therapist during internet-based treatment?
  ➔ Why would you like this/would you not like this?
  ➔ What do you think of complete self-help without the possibility of contact with a therapist?
  ➔ Why?
• Do you consider it possible to build a relationship with your therapist if you follow a treatment that is entirely internet-based, in which your therapist provides feedback on your exercises via chat or email?
  ➔ Why or why not?

d. Other

• What device would you prefer to use for following an internet-based treatment?
  ➔ Why do you prefer this?
  ➔ What are the advantages and disadvantages of the different options for you?
• What should an internet-based treatment platform look like?
